# Quantitative Phase Imaging Using Digital Holographic Microscopy to Assess the Degree of Intestinal Inflammation in Patients with Ulcerative Colitis

**DOI:** 10.3390/jcm12124067

**Published:** 2023-06-15

**Authors:** Arne Bokemeyer, Joost Buskermolen, Steffi Ketelhut, Phil-Robin Tepasse, Richard Vollenberg, Jonel Trebicka, Hartmut H. Schmidt, Michael Vieth, Dominik Bettenworth, Björn Kemper

**Affiliations:** 1 Department of Gastroenterology, Hepatology and Transplant Medicine, University Hospital of Essen, University Duisburg-Essen, 45147 Essen, Germany; arne.bokemeyer@uk-essen.de (A.B.); hartmut.schmidt@uk-essen.de (H.H.S.); 2 Department of Medicine B for Gastroenterology, Hepatology, Endocrinology and Clinical Infectiology, University Hospital Muenster, 48149 Muenster, Germany; joost.buskermolen@ukmuenster.de (J.B.); phil-robin.tepasse@ukmuenster.de (P.-R.T.); richard.vollenberg@ukmuenster.de (R.V.); jonel.trebicka@ukmuenster.de (J.T.); ced@innere-medizin.de (D.B.); 3 Biomedical Technology Center, University of Muenster, 48149 Muenster, Germany; ketelhut@uni-muenster.de; 4 Institut für Pathologie, Friedrich-Alexander-University Erlangen-Nürnberg, Klinikum Bayreuth, 95445 Bayreuth, Germany; michael.vieth@uni-bayreuth.de; 5 CED Schwerpunktpraxis Münster, 48149 Muenster, Germany

**Keywords:** quantitative phase imaging, digital holographic microscopy, inflammatory bowel disease, ulcerative colitis, inflammation, refractive index, histopathology

## Abstract

Ulcerative colitis (UC) is characterized by chronic inflammation of the colorectum. Histological remission has emerged as a potential future treatment goal; however, the histopathological assessment of intestinal inflammation in UC remains challenging with a multitude of available scoring systems and the need for a pathologist with expertise in inflammatory bowel disease (IBD). In previous studies, quantitative phase imaging (QPI) including digital holographic microscopy (DHM) was successfully applied as an objective method for stain-free quantification of the degree of inflammation in tissue sections. Here, we evaluated the application of DHM for the quantitative assessment of histopathological inflammation in patients with UC. In our study, endoscopically obtained colonic and rectal mucosal biopsy samples from 21 patients with UC were analyzed by capturing DHM-based QPI images that were subsequently evaluated using the subepithelial refractive index (RI). The retrieved RI data were correlated with established histological scoring systems including the Nancy index (NI) as well as with endoscopic and clinical findings. As a primary endpoint, we found a significant correlation between the DHM-based retrieved RI and the NI (R^2^ = 0.251, *p* < 0.001). Furthermore, RI values correlated with the Mayo endoscopic subscore (MES; R^2^ = 0.176, *p* < 0.001). An area under the receiver operating characteristics (ROC) curve of 0.820 confirms the subepithelial RI as a reliable parameter to distinguish biopsies with histologically active UC from biopsies without evidence of active disease as determined by conventional histopathological examination. An RI higher than 1.3488 was found to be the most sensitive and specific cut-off value to identify histologically active UC (sensitivity of 84% and specificity of 72%). In conclusion, our data demonstrate DHM to be a reliable tool for the quantitative assessment of mucosal inflammation in patients with UC.

## 1. Introduction

Ulcerative colitis (UC) is an idiopathic, chronic inflammatory disease affecting the colorectum and causing mucosal inflammation. It develops distally in the rectum, extends proximally in a continuous manner, and is characterized by its relapsing and remitting disease course [1,2]. Histologically, active UC is especially characterized by the presence of mucosal neutrophilic inflammation, more specifically within the lamina propria and surface epithelium, in the crypt epithelium (also known as cryptitis), as well as in the lumens of the crypts. Moreover, plasma cells located between colonic crypts and muscularis mucosae (known as basal plasmacytosis), an increase in lymphocytes and plasma cells within the lamina propria, surface epithelial damage (erosions and ulcers), crypt architectural distortions and atrophy, as well as a decrease in goblet cells (mucin depletion) can be found [3,4].

A treat-to-target approach is recommended to guide treatment regimens in inflammatory bowel disease (IBD). Clinical remission and endoscopic remission represent key treatment targets [2,5]. Besides the established therapeutic targets aiming for clinical remission and mucosal healing, histological healing is considered to be another promising endpoint for the treatment of UC [5,6]. Accumulating evidence shows that histological remission is associated with decreased hospitalization, decreased colectomy rates, and a decreased risk of developing colorectal carcinoma, whereas persistent inflammation is associated with increased relapse rates [7,8,9,10,11,12,13,14,15]. However, the histological assessment of tissue samples remains challenging: over 30 IBD-related histological scorings are available for the assessment of inflammatory tissue alterations [4,11,16,17,18] and extensive histopathological expertise in the field of IBD is frequently required for the correct application of these scores [4]. Therefore, an objective tool to quantify inflammation on a microscopic level is desirable.

Quantitative phase imaging (QPI) [19,20] such as digital holographic microscopy (DHM) [21] has been applied in earlier studies as a label-free, quantitative, and potentially automatable tool to assess tissue properties [22,23,24]. Technically, DHM measures the optical path length delay (OPLD) caused by a transparent specimen referenced to the surrounding medium. The OPLD can be used to derive the tissues’ refractive index (RI), which is related to the tissue density [22,23,25,26,27]. Moreover, DHM and related QPI technologies enable the OPLD-based quantification of the protein content within single cells and whole tissue samples [25,28] without artificial staining [19,20] and also reflect the contribution from extracellular compounds such as matrix proteins and collagen fibers [29].

Concerning IBD, DHM was successfully applied to quantify colonic inflammation in an experimental murine colitis model, where a strong correlation between the severity of inflammation and the RI was found [22]. Similarly, a significant difference in tissue RI data between patients with active Crohn’s disease (CD) and those in remission was observed [23]. In addition, DHM was shown to accurately assess fibrotic properties in colonic resections from CD-related strictures and was capable of differentiating stricturing and non-stricturing tissue samples [24]. Hence, QPI with DHM can be regarded as a promising tool to support clinicians and pathologists in the field of IBD in quantifying histological inflammation.

In this context, the aim of our study was to evaluate the application of DHM for the assessment of histopathological inflammation in tissue samples of patients with UC and to correlate DHM-based findings from the subepithelial RI with s standard histopathological evaluation using the validated Nancy index (NI) [30] in patients with UC. Secondary endpoints were obtained from the correlation of the subepithelial RI with other histological indices such as the Robarts histopathology index (RHI) [31], Geboes index [32], and Riley index [33], as well as with endoscopic examination, clinical symptoms, and laboratory values.

## 2. Materials and Methods

### 2.1. Experimental Design and Study Population

This prospective, monocentric study was performed at the Department of Medicine B, University Hospital Muenster, Germany and at the Biomedical Technology Center, University of Muenster, Germany. The study was conducted in accordance with the ethical guidelines of the 1975 Declaration of Helsinki and was approved by the Ethics Board of the University of Muenster and the Medical Council of Westphalia-Lippe, Germany (2017-476-f-S).

**Figure 1:**
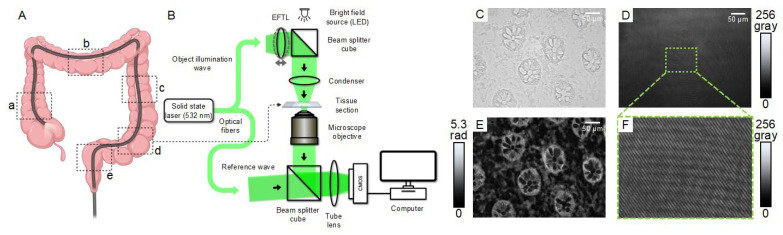
**Concept and experimental setup:** (**A**). During endoscopic examination, biopsies were taken in the ascending (**a**), transverse (**b**), descending (**c**), and sigmoid colon (**d**) and rectum (**e**) (see areas indicated with dashed rectangles), adapted from “Large Intestine (Colonoscopy)” by BioRender.com (2023) from https://app.biorender.com/biorender-templates, accessed on 24 March 2023; (**B**) Cryosections (thickness: 7 µm) from the biopsies were embedded in phosphate-buffered saline (PBS) and evaluated using a fiber optic Mach–Zehnder interferometer-based off-axis transmission DHM concept, utilizing an electrically focus tunable lens (EFTL) for modulation of the object illumination to achieve DHM QPI images with reduced coherence-induced image disturbances. CMOS: complementary metal-oxide semiconductor sensor, LED: light-emitting diode (adapted from [38]); (**C**) Bright field image of a representative biopsy; (**D**) Corresponding digital off-axis hologram. Gray: gray level; (**E**) Averaged quantitative phase image reconstructed from a series of 15 digital holograms as shown in (**D**). Rad: radian; (**F**) Enlarged part of the digital hologram that illustrates the off-axis carrier fringe interference pattern.

Patients with an established UC diagnosis and aged ≥18 years who underwent an ileocolonoscopy or a sigmoidoscopy between May 2019 and December 2021 were included in this study. Prior to inclusion, all patients provided informed consent. Mucosal biopsies of the ascending, transverse, descending, and sigmoid colon and rectum were obtained from those patients undergoing colonoscopy (Figure 1A). Amongst those patients who underwent a sigmoidoscopy, mucosal biopsies of the descending and sigmoid colon and rectum were obtained. For all patients, the Mayo endoscopic subscore (MES) [34] was documented per colon segment. Furthermore, blood samples were routinely collected prior to endoscopic examination. At the time of inclusion and 6 months later, clinical data including the partial Mayo score [35] were obtained during outpatient examinations. The exclusion criteria were as follows: aged below 18 years, unable to provide informed consent, and contraindications for endoscopic examination.

### 2.2. Histopathological Preparation

Directly after biopsy, the samples were transferred into 0.9% sodium chloride. Subsequently, they were embedded in O.C.T. compound (Optimal cutting temperature compound–Tissue Tek, Sukura Fine Tek Europe, Zoeterwoude, The Netherlands), shock frozen, and kept frozen at −80 °C until further use.

Tissue sections (thickness: 7 µm) were cut using a Cryostat-Microtome (MICROM HM550, Thermo Fisher Scientific, Waltham, MA, USA). From each sample, one cryosection was used for hematoxylin and eosin (H&E) staining and another cryosection was used for DHM analysis.

For H&E staining, cryosections were thawed, fixed in 70% ethanol for 2 min, and rinsed with distilled water before applying the hematoxylin solution (Sigma-Aldrich, Deisenhofen, Germany) for 5–10 min. Afterwards, the tissue was washed with tap water for 5–10 min and rinsed with distilled water. Subsequently, the eosin solution (Sigma-Aldrich, Deisenhofen, Germany) was applied for 2 min, followed by another rinsing with distilled water. Afterwards, the tissue was dehydrated with a series of increasing ethanol-concentrations and cleared with xylol before mounting with Vitro Clud (Langenbrinck, Emmendingen, Germany).

The H&E-stained tissue sections were then digitalized using a Nikon Eclipse Ni-E upright microscope (Nikon, Japan) and NIS-Elements AR Version 5.11.03 (Nikon, Japan). These digitalized images were examined by an IBD-specialized pathologist (MV) and used to identify regions of interest (ROI) for the DHM measurements. The IBD-specialized pathologist assessed several frequently used UC histopathological indices, with the validated NI being our primary score of interest. In addition, other indices were determined including the RHI, Geboes index, DCA [36], and Riley index.

### 2.3. Setup for Digital Holographic Microscopy, Sample Preparation, and Quantitative Phase Imaging

For the acquisition of brightfield images and off-axis digital holograms, an inverted Nikon Ts2R microscope (Nikon, Japan) with an attached fiber-optic Mach–Zehnder interferometer-based off-axis DHM module was applied utilizing a 20× microscope lens (Nikon Plan 20×/0.4, Nikon, Japan). The coherent light source for the recording of digital holograms was a fiber-coupled solid-state laser (Cobolt 06-DPL, λ = 532 nm, Cobolt AB, Solna, Sweden). Images and holograms were recorded using a complementary metal-oxide semiconductor (CMOS) sensor (UI-3260CP-M-GL, IDS Imaging Development Systems GmbH, Obersulm, Germany). Following earlier studies [37], by adequately aligning the light intensity ratio between the object and reference arm of the DHM setup, the contrast of the holographic off-axis carrier fringes was adjusted to a value ≥0.5, to minimize carrier fringe contrast-induced phase noise variations. Moreover, to reduce laser light-induced image disturbances, such as scattering and parasitic interference patterns, a series of 15 digital holograms were captured during each measurement while the amplitude and phase of the illumination laser light were modulated using an electrically focus tunable lens (EFTL, EL-10-30, Opto-tune, Switzerland), as described previously [38]. A schematic of the setup is shown in Figure 1B.

For bright field and DHM-based QPI imaging, cryostat sections were defrosted at room temperature for 2–5 min. The samples were embedded in 70 µL phosphate-buffered saline (PBS) and each tissue sample was covered with a 170 µm thick coverslip. To avoid over drying the sample, immediate analysis was performed. Depending on the quality of the tissue, 3 to 7 representative fields of views (FOVs) in the subepithelial mucosa were selected per cryostat section to record a series of 15 digital off-axes holograms for each FOV while the object wave was modulated. If it was not possible to acquire at least 3 hologram series of representative FOVs, the cryostat section was excluded from the analysis.

From the captured series of 15 digital holograms per FOV, quantitative phase images were reconstructed as described previously [39,40] using custom-built software (Biomedical Technology Center, University of Muenster, Germany) implemented in the programming environment PV-WAVE 9.5 (Rogue Wave Software, Inc., Louisville, Colorado, USA) which were subsequently averaged to achieve enhanced QPI [38]. Figure 1C–E shows a bright field image of a representative biopsy, a corresponding exemplary digital off-axis hologram, and the finally reconstructed averaged quantitative phase image of the sample. The averaged QPI images were then utilized to quantify the optical path length delay (OPLD) caused by the investigated cryostat sections in specific ROIs from which the RI, which is directly related to tissue density [22,23,25,26,27], was subsequently determined (see Section 2.4).

### 2.4. Determination of the Refractive Index in Mucosal Biopsies

The acquired averaged DHM QPI images (Figure 1E) were used to determine the OPLD using the open software ImageJ Version 2.3.0/1.53q (NIH, Bethesda, MD, USA). For each quantitative phase image, the mean OPLD was determined in 10 ROIs within the mucosal lamina propria (for illustration see Supplemental Figure S1). Typically, 3 to 7 FOVs per tissue section were analyzed, which resulted in a total of 30 to 70 analyzed ROIs per biopsy.

For mainly transparent tissue sections, such as the investigated cryostat sections, the sample-induced OPLD depends on the wavelength ***λ*** of the applied laser light (532 nm), the thickness ***d*_sample_** of the sample (7 µm), the sample refractive index ***RI*_sample_**, and the refractive index ***RI*_medium_** of the buffer medium (PBS) (1.337, determined using an Abbe Refractometer) [40]:

(1) $$\Delta \varphi \left(x,y\right)=\frac{2\pi }{\lambda }*d_{sample}*\left(RI_{sample}\left(x,y\right)-RI_{medium}\right).$$

Thus, for known parameters ***λ***, ***d*_sample_**, and ***RI*_medium_** from the sample-induced phase change $\Delta \varphi_{ROI}\left(x,y\right)$ the refractive index in an investigated ROI (***RI*_ROI_**) can be calculated:

(2) $$RI_{ROI}\left(x,y\right)=\left(\frac{\Delta \varphi_{ROI}\left(x,y\right)*\lambda }{d_{sample}*2\pi }\right)+RI_{medium}.$$

To determine the mean subepithelial *RI* of a tissue section the ***RI*_ROI_** values of all investigated ROIs were determined and subsequently averaged.

### 2.5. Statistical Analysis

SPSS Statistics Version 28.0.1.0 (IBM Corp., Armonk, NY, USA) was used for statistical analysis. The mean and SEM were determined for continuous variables, and frequencies and percentages are provided for categorical variables.

To calculate the Pearson correlation between the RI and histological scores, as well as for the correlation of RI and MES, values from all analyzed colon segments were used. For the correlation between the RI and blood values, as well as for the correlation between RI and clinical data, only the RI-data from rectal biopsies were used. The sigmoidal biopsy was used when the rectal biopsy was not of sufficient quality for analysis.

A receiver–operator characteristics (ROC) curve was created to illustrate the capability of the RI to predict histopathological active disease as determined by an NI > 1. The optimal cutoff point in terms of maximal sensitivity and specificity was determined using Youden’s index.

The Mann–Whitney U Test was used to detect differences between patients with UC in remission (either defined by the NI or by the RI) and patients with histopathological active UC.

When the Geboes index was used for statistical purposes, a six-point grading scale (grade 0–5) was used without taking subcategories into account (e.g., grade 1.1 is considered to be 1 and grade 2A.3 is considered to be 2).

Two-sided *p*-values < 0.05 were considered to be significant.

## 3. Results

### 3.1. Study Population

A total of 21 patients with UC undergoing endoscopic examination were included in the study. A fraction of 13/21 (62%) patients underwent a full (ileo-)colonoscopy and 8/21 (38%) underwent a partial colonoscopy up to the left colonic flexure. Exacerbation of clinical symptoms was the most common indication for endoscopic examination (9/21; 43%), followed by an evaluation of endoscopic response after therapeutical change (7/21; 33%) and surveillance colonoscopy (5/21; 24%; Table 1).

The mean age of the patients was 47.3 ± 3.3 years (Mean ± SEM). A total of 71% of patients were male and 29% were female. The average duration of disease at the time of inclusion was 127.4 ± 25.9 months. A majority of 13/21 (62%) patients suffered from pancolitis (extensity 3 (E3)) according to the Montreal Classification [41]), whereas 5/21 (24%) patients had left-sided colitis (E2) and 3/21 (14%) patients had proctitis (E1).

At the time of inclusion, patients had an average total Mayo score of 4.1 ± 0.8 points. The average Mayo endoscopic subscore (MES) for the most distal biopsy site, either rectal or sigmoidal, was 1.0 ± 0.2. The average NI at the corresponding biopsy site was 1.8 ± 0.4.

All patients except one (20/21; 95%) were treated with aminosalicylates. Additionally, 67% of patients with UC received an advanced medical therapy including monoclonal antibodies: more specifically, 9/21 (43%) patients were treated with tumor necrosis factor (TNF) inhibitors and 5/21 (24%) with vedolizumab, an α_4_β_7_ anti-integrin antibody. Furthermore, 3/21 (14%) patients were treated with corticosteroids. A more detailed overview considering medical treatment at the time of inclusion can be found in Table 1. None of the patients previously underwent surgery on their colon.

### 3.2. Microscopically Analyzed Biopsies

**Figure 2:**
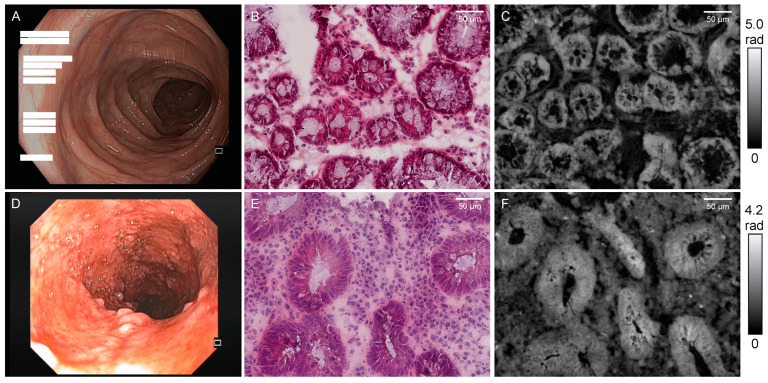
Representative endoscopy (**A**,**D**), H&E-staining (**B**,**E**), and DHM-QPI (**C**,**F**) images from a patient with UC in remission (upper row, (**A**–**C**)) as well as from a patient with active UC (lower row, (**D**–**F**)). Rad: radian.

A total number of 78 colonic biopsies from 21 patients were prepared as described in Section 2.2 and evaluated using both DHM (Section 2.3 and Section 2.4) and common histopathological analysis (Figure 2). Histological evaluation yielded an average NI of 1.44 ± 0.20 (Mean ± SEM), an average Geboes index of 1.23 ± 0.21, an average RHI of 4.36 ± 0.54, and an average Riley index of 1.91 ± 0.16. The corresponding average Mayo endoscopic subscore (MES) was 0.67 ± 0.09.

Analysis of a total of 404 DHM-QPI images resulted in an average RI per biopsy of 1.3490 ± 0.0003 (Mean ± SEM).

### 3.3. Correlation of Refractive Index with Histological Scorings and Endoscopic Findings

**Figure 3:**
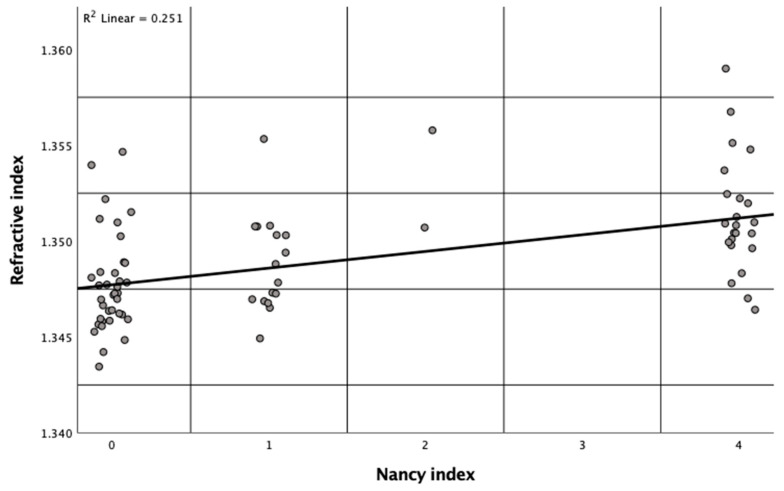
Significant correlation (*p* < 0.001) between the subepithelial refractive index as determined by digital holographic microscopy and the Nancy index as determined by histopathological examination in 78 biopsies from patients with ulcerative colitis. One dot represents the data of one biopsy.

The RI in mucosal biopsies of the colon as measured by QPI significantly correlates with histopathological inflammation as determined by NI (R^2^ = 0.251, *p* < 0.001). The plot in Figure 3 shows that higher RI data among 78 biopsies correlate with higher NI values. Similar correlations (Supplemental Table S1) were found between the RI and other established histological scorings for inflammatory tissue alterations in patients with UC including the Geboes index (R^2^ = 0.232, *p* < 0.001), the RHI (R^2^ = 0.219, *p* < 0.001), and the Riley index (R^2^ = 0.223, *p* < 0.001).

**Figure 4:**
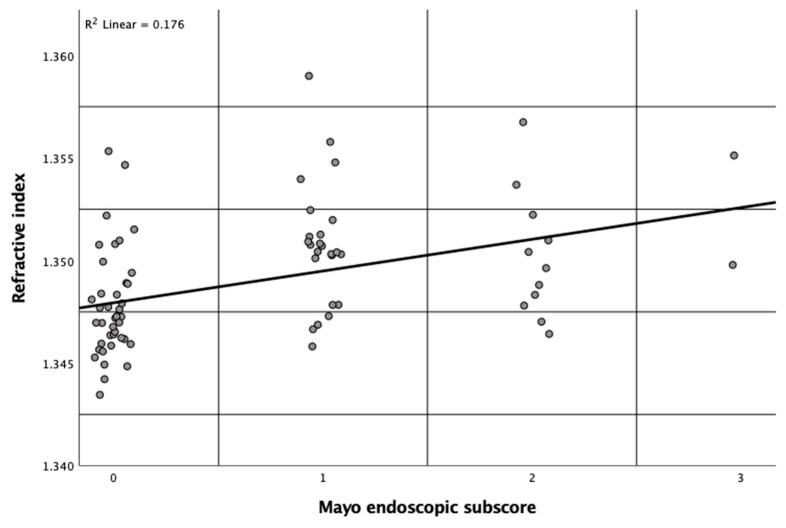
Significant correlation (*p* < 0.001) between the subepithelial refractive index as determined by digital holographic microscopy in 78 biopsies and Mayo endoscopic subscore in 78 corresponding colon segments from patients with ulcerative colitis. One dot represents the data of one biopsy and its corresponding biopsy site.

In line with the correlation between RI data and histopathological scorings, the plot in Figure 4 shows that the RI significantly correlates with the MES (R^2^ = 0.176, *p* < 0.001), indicating that higher RI values positively correlate with endoscopically observed disease activity.

### 3.4. Capability of Digital Holographic Microscopy to Distinguish Ulcerative Colitis in Remission from Active Disease

**Figure 5:**
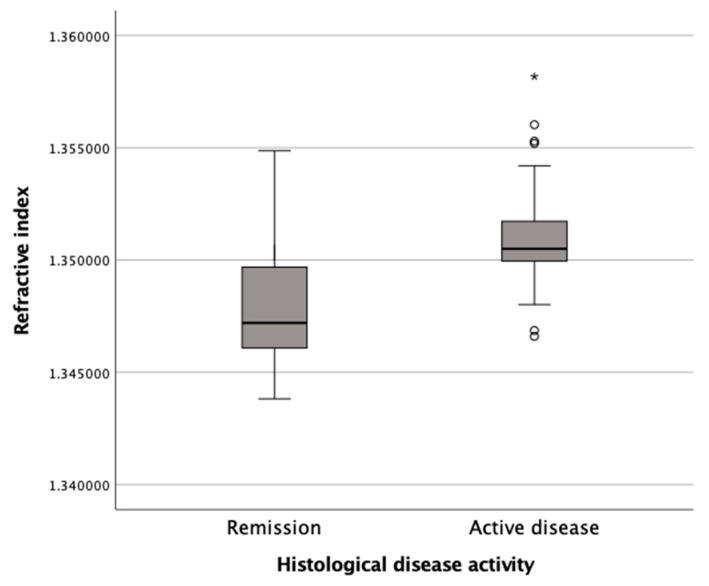
The subepithelial refractive index in colonic mucosal biopsies (n = 78) as determined by digital holographic microscopy was significantly lower (* *p* < 0.001) in biopsies taken from patients with ulcerative colitis in histological remission as determined by a Nancy index ≤ 1 compared to biopsies with histological signs of ulcerative colitis disease activity (NI > 1).

The subepithelial RI as determined by DHM allowed us to reliably distinguish colonic and rectal mucosal biopsies with histological disease activity from those in remission. As plotted in Figure 5, colonic and rectal biopsies with histologically active disease, as defined by an NI higher than 1, showed a significantly (*p* < 0.001) higher average RI of 1.3512, ± 0.0029 (Mean ± SEM) compared to biopsies with signs of histological remission (1.3479 ± 0.0025) with an NI of 0–1.

**Figure 6:**
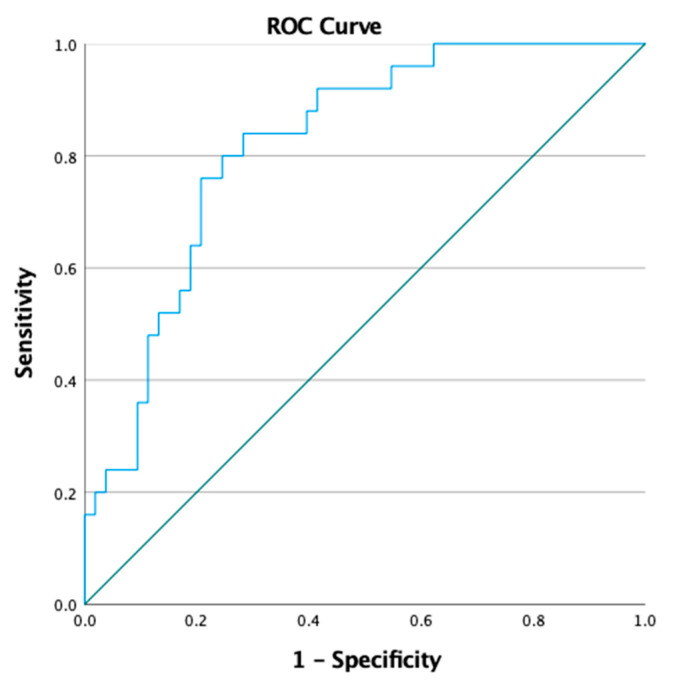
The receiver–operator characteristics (ROC) curve with an area under the curve (AUC) of 0.820 shows that the subepithelial refractive index as determined by digital holographic microscopy is a reliable parameter to distinguish colonic mucosal biopsies (n = 78) from patients with ulcerative colitis in histological remission as determined by a Nancy index ≤ 1 from biopsies with histological signs of ulcerative colitis disease activity (NI > 1). The ROC curve was retrieved considering the data shown in Figure 5.

Consequently, the receiver–operator characteristics (ROC) curve with an area under the curve (AUC) of 0.820 (Figure 6) illustrates that the RI can be used to reliably distinguish biopsies with histologically active disease (NI > 1) from biopsies with disease in remission. Using the Youden’s index, an RI of 1.3488 was found to be the most sensitive and specific cut-off value to distinguish both groups and had a sensitivity of 84% and a specificity of 72% to diagnose active histopathological disease amongst patients with UC; a biopsy with an RI > 1.3488 is likely to have histological signs of active disease (NI > 1).

### 3.5. Correlations of the Refractive Index with Laboratory Inflammation Markers and Clinical Findings

No correlation was found between the subepithelial RI in rectal or sigmoidal biopsies (n = 21, only the most distal biopsy was used for this analysis) and laboratory inflammatory markers (Supplemental Table S2) including the c-reactive protein (CRP) (*p* = 0.61) and white blood cell count (*p* = 0.20). Likewise, no correlation was found between the RI and fecal calprotectin (*p* = 0.42). Fecal calprotectin values were available in only 5/21 patients. A tendency towards a positive correlation between the RI and the clinical mayo score was observed; however, this did not reach statistical significance (*p* = 0.09).

Patients with UC with an RI > 1.3488 at the most distal biopsy site had a significantly higher total Mayo score of 5.3 ± 3.4 (Mean ± SEM) compared to a Mayo score of 1.9 (SEM ± 3.3) for those with a lower RI (*p* < 0.05, Supplemental Figure S2), which again demonstrates the ability of the refractive index to distinguish between active disease and remission.

## 4. Discussion

To the best of our knowledge, our study evaluates the application of DHM for the quantification of inflammation in human colon samples from patients with UC for the first time. We found a significant correlation between the subepithelial RI and the NI (Figure 3) and between the subepithelial RI and MES (Figure 4). Our data demonstrate that the tissue RI is significantly higher in biopsies with signs of active UC compared to biopsies with an NI ≤ 1 (Figure 5). Moreover, the tissue RI can reliably distinguish patients with UC with histological remission from those with histologically active disease (Figure 6).

Current IBD guidelines state that mucosal healing, assessed using endoscopy, is the desired long-term treatment goal in patients with UC [2,5,42]. However, in addition to mucosal healing, histopathological healing might become the next treatment target in patients with UC [5]. A recent meta-analysis demonstrated that histological healing in addition to mucosal healing is prognostically favorable for patients with UC: patients with both mucosal and histological healing had only a 5% annual risk of clinical relapse compared to an annual clinical relapse risk of 14% for patients with UC with mucosal healing who did not achieve histological remission [15]. In another large meta-analysis, patients with UC in endoscopic remission had higher clinical relapse rates when they had persistent histological activity [43]. Moreover, a recent cohort study found a significantly lower hospitalization and surgery risk among patients with UC with histological disease clearance [12]. Another trial showed that patients with UC with histological healing (NI ≤ 1) had significantly longer clinical relapse-free survival compared to patients with histological active disease (NI > 1) [14].

Considering histological healing as the upcoming therapeutic target [5,44], accurate histopathological analysis might become a key feature in the guidance of IBD therapy. Therefore, an efficient, time-saving, and reliable evaluation of histological remission is essential [4]. In the field of IBD, a multitude of over thirty different scoring systems to classify histopathological inflammation are available [4,23], of which two have been recently validated for UC and are recommended by European guidelines [3]. One of the validated scoring systems is the Nancy index. It defines five grades of disease activity: the absence of any significant histological disease (grade 0, remission), chronic infiltrate without mucosal neutrophils (grade 1, histological response), mild active disease with only a few neutrophils (grade 2), moderate to severe neutrophil infiltrates (grade 3), and ulceration (grade 4) [30,45]. The second validated scoring system is the Robarts histopathology index [31], which is based on the Geboes score and considers several items including signs of chronic inflammation of the lamina propria, neutrophils in the lamina propria, epithelial neutrophils, and surface epithelial injury. Despite the development of the RHI, the Geboes score is still frequently used in daily clinics and current medical trials [32,46].

Although histological assessment with the establishment of validated scoring systems, as described above, has made progress during the last years [3], many histological scoring systems do not consider all relevant aspects of IBD pathology, for example, basal plasmacytosis [16,17,23] and histological assessment as well as the fact that correct the usage of scoring systems requires highly experienced IBD pathologists [16,47]. Furthermore, it was recently shown that knowledge of IBD histological scoring systems and their utilization is poor amongst gastroenterologists and pathologists, despite recognition of their importance [48]. Therefore, quantitative, reliable, time-efficient, and automatable tools in terms of digital pathology are desired, which might be capable of assessing histological inflammation independently, as an additional support to the classic histological assessment that is performed by a pathologist.

QPI as provided by DHM could potentially fill this gap due to some unique features: The technology is label-free and, thereby, avoids artificial staining of tissue sections [22,27,49,50]. Moreover, it enables the quantification of tissue density based on refractive index determination with minimized sample preparation efforts [22,51,52]. Park et al. demonstrated that QPI offers quantitative information in an automated manner in the diagnosis of clear-cell renal cell carcinoma [53], a disease that is difficult to diagnose using common microscopic histopathological evaluation. Based on our findings, QPI could play a similar role when it comes to quantifying inflammation on a microscopic level in patients with UC. Subepithelial RI data as determined by QPI with DHM correlated with several well-established pathological indices such as the NI (R^2^ = 0.251) as well as with endoscopic findings (R^2^ = 0.176), as plotted in Figure 3 and Figure 4, respectively. Moreover, an area under the ROC curve of 0.820 (Figure 6) suggests that the RI is an excellent parameter for distinguishing patients with UC with histological remission/response (NI ≤ 1) from those with histologically active disease (NI > 1). This is supported by the finding that patients with an RI > 1.3488 at the most distal biopsy site (rectum or sigmoid) had a significantly higher Mayo score compared to patients with a lower RI at the most distal biopsy site (Supplemental Figure S2).

Previously, we evaluated the use of DHM to assess inflammatory alterations in single-cell systems and colonic tissue sections [22,23,24,26]. In an experimental murine colitis model, the RI was significantly reduced in all layers of the colonic wall compared to healthy controls, likely as a consequence of a chemical-induced acute edema [22]. In contrast, in our current findings in samples from human patients with UC we observed a positive correlation between RI data and endoscopic and histological inflammation (Figure 3 and Figure 4). This could be explained by cell infiltrates in tissue samples with active UC (Figure 1E) [4,18,54], which is supported by the correlation with several IBD scoring systems that include the degree of cell infiltration as a critical component of histological evaluation [30,31,32,33].

Compared with our previous studies [22,24,26] we found, on average, slightly lower absolute RI values (results Section 3.2). A technical explanation for this result is that our current study was conducted using a revised experimental DHM setup. The setup consisted of a different microscope embodiment for optical imaging and also included a sample illumination modulation-based method to achieve enhanced QPI by reducing laser light-induced image disturbances [38], such as scattering and parasitic interference patterns, which can cause overestimated OPLD values and, thus, increased RI data, respectively. Moreover, variations in the tissue section thickness can induce changes in light scattering properties and QPI image quality [20]. This can affect the RI retrieval with Equation (2) and has to be considered, in particular, when experimental data from different laboratories are compared. On the one hand, thin tissue sections cause, in the case of samples with small spatial refractive index variations, a decreased signal-to-noise ratio and QPI image disturbances due to increased mechanical tissue damage during the cutting process. On the other hand, a tissue section thickness that is higher than a few cell layers induces increased scattering of the illuminating laser light, which results in increased phase noise and can cause artifacts during the numerical reconstruction of the QPI images. To limit the impact of differences in thickness and quality of the prepared cryosections and accompanied potential image disturbances and uncertainties in the RI determination with Equation (2), a reproducible standardized sample preparation is essential. Thus, in our study, all cryo-sectioning and sample preparation steps were conducted in the same manner and by the same investigators.

In addition to the utilization of DHM for the assessment of biopsy samples for histopathological characterization, it would significantly support the direct guidance of IBD therapy, if DHM could be used in vivo during endoscopic examination. Other groups have already started exploring QPI in the in vivo setting: Hu et al. developed an endoscopic QPI system by attaching a diffraction phase microscopy module to an endoscope probe, which allowed single-shot endoscopic phase imaging with cell-level resolution [55]. Gordon et al. demonstrated the detection of biologically relevant quantitative phase properties from early tumors in esophageal tissue using holographic endomicroscopy [56]. Similarly, in [57] Costa et al. report a fiber-based handheld QPI device based on quantitative oblique back illumination microscopy (qOBM) for in vivo intraoperative use. Hence, QPI potentially could offer information on the state of histological healing in vivo during endoscopic examination.

The limited number of patients and the monocentric setting of our study restricts the generalization of our conclusion to other patient populations. However, due to the prospective study design, we could acquire a complete data set strengthening the value of our results. Despite the limited patient number, we found a statistical significance in the correlation between the subepithelial RI and histological indices, stressing the possibilities that QPI with DHM offers. However, a multicenter study with a larger sample size evaluating DHM amongst patients with UC would be desirable and could provide advanced insights, for example, into the RI response to medical treatment.

Secondly, to increase the power of our study, multiple biopsies from the same patient were used. These biopsies were taken from different colon segments with varying degrees of inflammation (e.g., normal mucosa in the ascending colon and severely inflamed mucosa in the rectum from the same patient). Although all colonic segments were individually assessed using endoscopy, DHM, and histopathological analysis, a certain degree of ‘interdependence’ cannot be ruled out entirely.

Thirdly, the study was limited to patients with UC and did not address other types of colitis such as infectious colitis. However, the results of our study build the basis for future in-depth investigation on the evaluation of QPI in the entire spectrum of colorectal inflammatory processes.

In conclusion, the results of our study suggest DHM-based QPI as a powerful and efficient method to objectively assess the degree of histological inflammation in patients with UC in a potentially automated manner and as a promising tool to support pathologists in terms of digital pathology.

## Figures and Tables

**Table 1:** Characteristics of the patients with an established ulcerative colitis diagnosis who underwent an ileocolonoscopy or a sigmoidoscopy that were included in the study. For continuous variables the mean and standard error of mean (SEM) are provided, while for categorical variables frequencies and percentages are reported.

Variables	Patients (N = 21)
Age in years (Mean ± SEM)	47.3 ± 3.3
Female	6 (29%)
Male	15 (71%)
Patients undergoing a colonoscopy	13 (62%)
Patients undergoing a sigmoidoscopy	8 (38%)
Duration of disease in months (Mean ± SEM)	127.4 ± 26.9
*Indication for examination*	
Colorectal dysplasia screening	5 (24%)
Control examination after therapy change	7 (33%)
Exacerbation of clinical symptoms	9 (43%)
*Disease activity*	
Average Mayo endoscopic subscore at most distal biopsy site (Mean ± SEM)	1.0 ± 0.2
Average Mayo score (Mean ± SEM)	4.1 ± 0.8
Average Nancy index at most distal biopsy site (Mean ± SEM)	1.8 ± 0.4
*Disease localization according to Montreal Classification*	
Extensity 1; ulcerative proctitis	3 (14%)
Extensity 2; left-sided UC	5 (24%)
Extensity 3; pancolitis	13 (62%)
*Medical treatment*	
*Biologics*	
Antitumor necrosis factor agents (Adalimumab, Infliximab, and Golimumab)	9 (43%)
Integrin α4β7 antagonist (Vedolizumab)	5 (24%)
*Immunosuppressants*	
Azathioprine	2 (10%)
Cyclosporine A	1 (5%)
*Small molecules*	
Tofacitinib	1 (5%)
*Corticosteroids*	
Oral	1 (5%)
Rectal	2 (19%)
*Aminosalicylates*	
Mesalazine oral	15 (71%)
Mesalazine rectal	4 (19%)
Sulfasalazine oral	1 (5%)

## Data Availability

The original datasets are available from the corresponding author on reasonable request.

## Supplementary Materials

The following supporting information can be downloaded at https://www.mdpi.com/article/10.3390/jcm12124067/s1, Figure S1: Exemplary image for the determination of subepithelial ROIs; Figure S2: Significant higher total Mayo score 5.3 ± 3.4 (Mean ± SEM) amongst patients with ulcerative colitis with an RI > 1.3488 in the most distal biopsy compared to a Mayo score of 1.9 (SEM ± 3.3) for those with a lower RI at the most distal biopsy site (*p* < 0.05); Table S1: Significant correlations between tissue refractive index as determined by using a digital holographic microscopy and multiple histopathological in 78 biopsies from patients with ulcerative colitis; and Table S2: Pearson correlations between tissue refractive index as determined by using a digital holographic microscopy and clinical data at the most distal biopsy site (n = 21).
