# Supplementary material for: Quantitative Phase Imaging Using Digital Holographic Microscopy to Assess the Degree of Intestinal Inflammation in Patients with Ulcerative Colitis
Source: J Clin Med. 2023 Jun 15;12(12):4067. doi: 10.3390/jcm12124067 (PMC10299047; doi:10.3390/jcm12124067)
Supplement: Supplementary file 1 [file jcm-12-04067-s001.zip › jcm-2376744-supplementary.pdf]

Figure S1: Exemplary image for the determination of subepithelial ROIs.

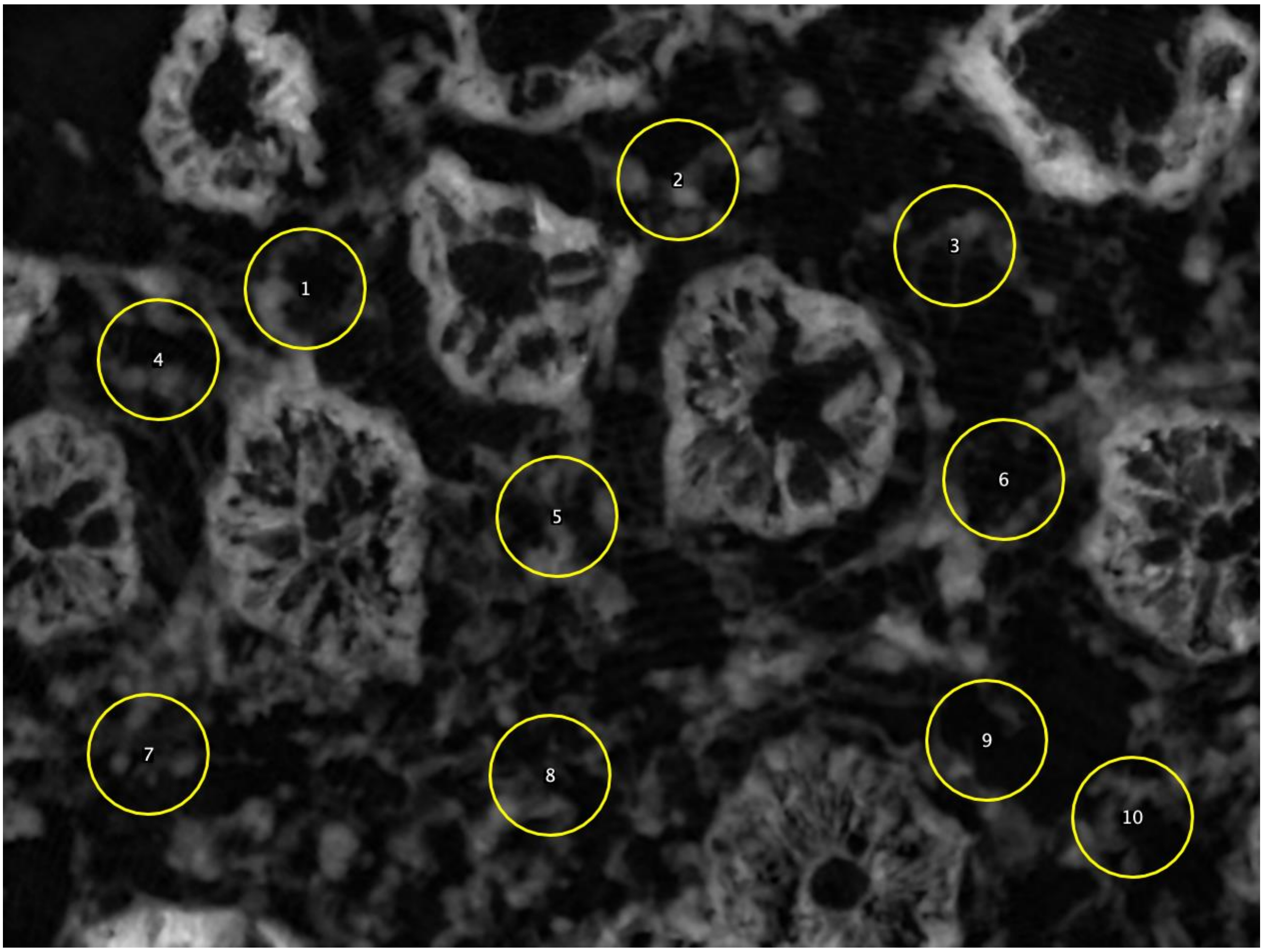

Figure S2: Significantly higher total Mayo score 5.3 ± 3.4 (Mean ± SEM) amongst UC patients with a RI > 1.3488 in the most distal biopsy compared to a Mayo score of 1.9 (SEM ± 3.3) for those with a lower RI at the most distal biopsy site ($p<0.05$).

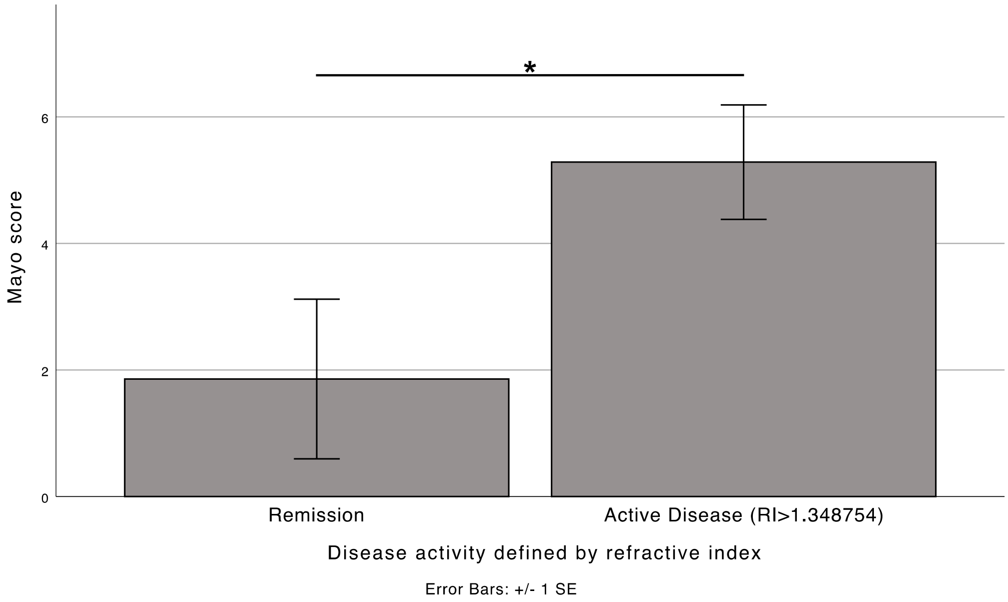

Table S1: Significant correlations between tissue refractive index as determined by digital holographic microscopy and multiple histopathological in 78 biopsies from patients with ulcerative colitis.

Correlations

		Refractive index	Nancy Index	GEBOES	RHI	Riley	Endoscopic Mayo-Score
Refractive index	Pearson Correlation	1	.501**	.482**	.468**	.472**	.419**
	Sig. (2-tailed)		<0.001	<0.001	<0.001	<0.001	<0.001
	N	78	78	78	78	78	78

**. Correlation is significant at the 0.01 level (2-tailed).

Table S2: Pearson correlations between tissue refractive index as determined by digital holographic microscopy at the most distal biopsy site (n=21) and clinical data/laboratory values.

Correlations

		Refractive index
Refractive index	Pearson Correlation	1
	Sig. (2-tailed)	
	N	23
CRP in mg/dl	Pearson Correlation	0.121
	Sig. (2-tailed)	0.613
	N	20
Leukocytes in tsd/microl	Pearson Correlation	0.289
	Sig. (2-tailed)	0.204
	N	21
Thrombocytes in tsd/microl	Pearson Correlation	0.095
	Sig. (2-tailed)	0.682
	N	21
Faecal Calprotectin in mg/kg	Pearson Correlation	0.470
	Sig. (2-tailed)	0.424
	N	5
Stool Frequency	Pearson Correlation	0.369
	Sig. (2-tailed)	0.100
	N	21
Rectal Bleeding	Pearson Correlation	0.143
	Sig. (2-tailed)	0.537
	N	21
Endoscopic score	Pearson Correlation	0.413
	Sig. (2-tailed)	0.063
	N	21
Physician Assessment	Pearson Correlation	0.417
	Sig. (2-tailed)	0.060
	N	21
Mayo score	Pearson Correlation	0.378
	Sig. (2-tailed)	0.091
	N	21

*. Correlation is significant at the 0.05 level (2-tailed).

**. Correlation is significant at the 0.01 level (2-tailed).
